# Biomarkers of sustained systemic inflammation and microvascular dysfunction associated with post-COVID-19 condition symptoms at 24 months after SARS-CoV-2-infection

**DOI:** 10.3389/fimmu.2023.1182182

**Published:** 2023-10-05

**Authors:** Lotte M. C. Jacobs, Marieke S. J. N. Wintjens, Magdolna Nagy, Loes Willems, Hugo ten Cate, Henri M. H. Spronk, Sander M. J. van Kuijk, Chahinda Ghossein-Doha, Mihai G. Netea, Laszlo A. Groh, André S. van Petersen, Michiel C. Warlé

**Affiliations:** ^1^ Department of Surgery, Radboud University Medical Center, Nijmegen, Netherlands; ^2^ Department of Clinical Epidemiology and Medical Technology Assessment, Maastricht University Medical Center+ (UMC+), Maastricht, Netherlands; ^3^ Department of Intensive Care Medicine, Maastricht University Medical Center+ (UMC+), Maastricht, Netherlands; ^4^ Department of Biochemistry, Maastricht University Medical Center+ (UMC+), Maastricht, Netherlands; ^5^ Cardiovascular Research Institute Maastricht (CARIM), Maastricht University, Maastricht, Netherlands; ^6^ Department of Internal Medicine, Maastricht University Medical Center+ (UMC+), Maastricht, Netherlands; ^7^ Center for Thrombosis and Haemostasis, Gutenberg University Medical Center, Mainz, Germany; ^8^ Department of Cardiology, Maastricht University Medical Center+ (UMC+), Maastricht, Netherlands; ^9^ Department of Internal Medicine and Radboud Center for Infectious Diseases, Radboud University Medical Center, Nijmegen, Netherlands; ^10^ Department of Immunology and Metabolism, Life & Medical Sciences Institute, University of Bonn, Bonn, Germany; ^11^ Department of Molecular Cell Biology and Immunology, Amsterdam Cardiovascular Sciences, Amsterdam Gastroenterology Endocrinology Metabolism, Amsterdam Institute for Infection and Immunity, Cancer Centre Amsterdam, Amsterdam University Medical Center (UMC), Vrije Universiteit Amsterdam, Amsterdam, Netherlands; ^12^ Department of Surgery, Bernhoven Hospital, Uden, Netherlands

**Keywords:** SARS-CoV-2, post-COVID-19 condition, coagulation activation, microvascular dysfunction, inflammation

## Abstract

**Introduction:**

Comprehensive studies investigating sustained hypercoagulability, endothelial function, and/or inflammation in relation to post-COVID-19 (PCC) symptoms with a prolonged follow-up are currently lacking. Therefore, the aim of this single-centre cohort study was to investigate serum biomarkers of coagulation activation, microvascular dysfunction, and inflammation in relation to persisting symptoms two years after acute COVID-19.

**Methods:**

Patients diagnosed with acute SARS-CoV-2 infection between February and June 2020 were recruited. Outcome measures included the CORona Follow-Up (CORFU) questionnaire, which is based on an internationally developed and partially validated basic questionnaire on persistent PCC symptoms. Additionally, plasma biomarkers reflecting coagulation activation, endothelial dysfunction and systemic inflammation were measured.

**Results:**

167 individuals were approached of which 148 (89%) completed the CORFU questionnaire. At 24 months after acute infection, fatigue was the most prevalent PCC symptom (84.5%). Over 50% of the patients experienced symptoms related to breathing, cognition, sleep or mobility; 30.3% still experienced at least one severe or extreme (4 or 5 on a 5-point scale) PCC symptom. Multiple correlations were found between several PCC symptoms and markers of endothelial dysfunction (endothelin-1 and von Willebrand factor) and systemic inflammation (Interleukin-1 Receptor antagonist). No positive correlations were found between PCC symptoms and coagulation complexes.

**Discussion:**

In conclusion, this study shows that at 24 months after acute COVID-19 infection patients experience a high prevalence of PCC symptoms which correlate with inflammatory cytokine IL-1Ra and markers of endothelial dysfunction, especially endothelin-1. Our data may provide a rationale for the selection of treatment strategies for further clinical studies.

**Trial registration:**

This study was performed in collaboration with the CORona Follow-Up (CORFU) study (NCT05240742, https://clinicaltrials.gov/ct2/show/ NCT05240742).

## Introduction

The coronavirus disease 2019 (COVID-19) pandemic caused by severe acute respiratory syndrome coronavirus 2 (SARS-CoV-2) has resulted in substantial mortality and morbidity worldwide (1). Shortly after the announcement of the global pandemic in March 2020 by the World Health Organization (WHO) (2), hardly anyone could suspect that this infection could lead to a chronic disease (3). A systematic review investigating persistent symptoms revealed that a median proportion of 72.5% of previously infected individuals experienced at least one persistent symptom up to 8 months post infection (4). The WHO developed a clinical case definition of post-COVID-19 condition (PCC): ‘*Post COVID-19 condition occurs in individuals with a history of probable or confirmed SARS CoV-2 infection, usually 3 months from the onset of COVID-19 with symptoms and that last for at least 2 months and cannot be explained by an alternative diagnosis’* (5).

In contrast to PCC symptoms, biomarkers associated with PCC have been scarcely investigated and the pathogenesis of PCC is largely unknown (6). Previous results from our group revealed that at 3 months after acute COVID-19, there was sustained coagulation activation, endothelial dysfunction, and systemic inflammation (7). Another study reported that at 6-12 weeks after COVID-19 infection no association was found between the most prevalent PCC symptom fatigue and the pro-inflammatory cytokine interleukin (IL)-6 (8). Comprehensive studies investigating sustained hypercoagulability, endothelial function, and/or inflammation in relation to PCC symptoms with a prolonged follow-up are currently lacking. Therefore, the main objective of this study was to investigate plasma biomarkers of coagulation activation, microvascular dysfunction, and inflammation in relation to PCC symptoms two years after acute infection.

## Materials and methods

### Study design

This cohort study was initiated at Radboud University Medical Center (Radboudumc, Nijmegen, the Netherlands) in collaboration with the National CORona Follow-Up (CORFU) study (NCT05240742) in the Netherlands and conducted at Bernhoven Hospital (Uden, the Netherlands) from November 2021 until July 2022. It was approved by the regional ethics committee Arnhem-Nijmegen (NL74101.091.20) and local approval was granted by the local directory boards. Additionally, for the CORFU study, ethics approval was obtained from the medical research ethics committee (MREC) of Maastricht University Medical Center+ and Maastricht University (METC2021-2990). Determinations and data handling were performed in agreement with the guidelines of The National Institutes of Health and in accordance with the declaration of Helsinki and its later amendments.

### Study population

In this study, patients who previously participated in a cohort study were contacted (7), including 203 patients diagnosed with acute SARS-CoV-2 infection between March and June 2020. All patients with SARS-CoV-2 infection confirmed by polymerase chain reaction during the acute infection on a nasopharyngeal swab, sputum or bronchoalveolar lavage at Bernhoven Hospital between March 1^st^ and June 1^st^ were given written information about the study. Patients were contacted for participation in the original study if they indicated they wanted to be approached. For the current study, blood was drawn 18-24 months after acute SARS-CoV-2 infection for measurement of biomarkers in plasma and participants were asked to fill in a questionnaire regarding PCC symptoms. Additionally, ten healthy volunteers were included as controls. Healthy volunteers were included in autumn 2020 if they had neither experienced any symptoms of COVID-19 infection nor had been in recent contact with anyone who was infected with COVID-19. Written informed consent was obtained from the participants before the start of any study-related procedures.

### Determining the prevalence of PCC symptoms

All participants were approached 24 months after COVID-19 infection to respond to the CORFU questionnaire on long COVID outcomes and determinants that was developed by the EuroQol Research Foundation (9). The CORFU questionnaire was based on an internationally developed basic questionnaire on persistent symptoms after COVID-19. In case of limited understanding of computers, participants were offered the option to receive the survey by mail. The primary outcome was the prevalence of PCC complaints.

### Measurement of Endothelin-1, inflammatory cytokine concentrations,and coagulation complexes

A whole blood sample was obtained by venipuncture in Lithium-Heparin (Vacuette) tubes (10 mL) during the study visit at 18-24 months after SARS-CoV-2 infection. Blood samples were centrifugated at 2500 g for 10 minutes at room temperature, followed by a second centrifugation step at 2500 g for 20 minutes at room temperature. Subsequently, the plasma was stored at -80 °C until further analysis. Plasma concentrations of Endothelin-1 (ET-1), interleukin-18 (IL-18), interleukin-6 (IL-6), and interleukin-1 receptor antagonist (IL-1Ra) were determined batchwise using R&D Systems Quantikine^®^ ELISA Kits (R&D, Minneapolis, Minnesota, catalogue numbers DET100, DL180, D6050 and DRA00B, respectively) according to the manufacturer’s protocol. Levels of activated coagulation factors in complex with their natural inhibitors [thrombin:antithrombin (TAT), factor(F)IXa : AT, FVIIa : AT, FXIa : AT, FXIa:alpha-1-antitrypsin(α1AT), FXIa:C1-esterase-inhibitor(C1inh)] and von Willebrand factor antigen (VWF : Ag) were quantified using in-house developed enzyme-linked immunosorbent assays (ELISAs) (10). For determining the VWF : Ag levels, rabbit anti-human vWF antibody (1 in 1500, Dako A082) was used as capture antibody, whereas HRP-labeled rabbit anti-human VWF antibody (1 in 5000, Dako P226) was applied as detection antibody. The VWF : Ag level in individual samples was determined using a standard curve made of standard human plasma (Siemens Healthineers) with known concentration of vWF : Ag.

Normal ranges of in-house developed ELISA methods were defined as above normal mean ± 1 standard deviation (SD), based on previous validation studies (7, 10). Additionally, the normal ranges of inflammatory cytokines were compared to literature regarding levels in healthy controls and diseased patients and were adjusted if necessary (11–13).

### Measurement of plasma protein concentrations

Circulating plasma protein expression for 23 PCC subjects (only the participants who also consented with blood plasma preservation during the acute phase of the infection (7)), with samples collected during the acute phase of the infection, 3 months after recovery (7), and 18-24 months after recovery, and 10 healthy volunteers were assessed using the commercially available multiplex proximity extension assay from Olink^®^ Proteomics AB (Uppsala Sweden). The Target 96 Inflammation Panel was run where 96 inflammatory proteins were measured. Statistical testing was performed by using the Wilcoxon matched-pairs signed rank test. For volcano plots, data were analysed by Mann-Whitney test and the Benjamini-Hochberg procedure was employed to correct for multiple testing errors. False discovery rate (FDR)-adjusted p-values smaller than 0.05 were considered statistically significant. Statistical analysis and data visualization were performed with R/Bioconductor (https://www.R-project.org/).

### Statistical analysis

Values were reported as mean ± SD, median (IQR) or n (%). Changes in biomarkers between 18-24 months and 3 months [as previously measured (7)] were tested using the Wilcoxon Signed Rank Test. Spearman’s *r* was calculated to examine correlations between PCC complaints, coagulation factors, markers of endothelial dysfunction and inflammatory cytokines. Linear regression with backward elimination of variables was performed to adjust for initial disease severity, comorbidities and baseline characteristics. Total symptoms score was calculated for each patient as the sum of PCC symptoms with at least moderate severity. Differences in PCC symptoms and biomarkers between patients who recovered at home, were hospitalised, or were transferred to the intensive care unit were determined by ANOVA. For plasma cytokine levels, also the proportion of patients with concentrations above normal range was reported. Figures were made using Graphpad Prism version 9.4.1 and statistical analyses were performed using IBM SPSS Statistics 27. P-values below 0.05 were considered statistically significant.

## Results

### Study population

Two hundred three patients who visited Bernhoven hospital for the COVAS trial at approximately 3 months after COVID-19 infection were contacted for the study visit. 36 participants did not participate in this study due to loss to follow-up, withdrawal of consent, or death. This resulted in a total of 167 participants who were approached to fill in the questionnaire at 24 months after COVID-19 infection. 148 participants (89%) completed the survey (**Supplementary Figure 1**). Patient characteristics are shown in **Table 1**. Furthermore, 10 healthy controls (3 males and 7 females with mean age ± SD: 35.6 ± 11.1) were included for Olink analyses.

**Table 1:** Characteristics of all participants who completed the post-COVID symptoms questionnaire.

		T1 + T2 (n = 148)	T0 + T1 + T2 (n = 23)
**Patient characteristics**	Age, mean (SD)	64.1 (11.6)	65.7 (12.6)
	Male sex, N (%)	96 (64.0)	15 (65.2)
	BMI, mean (SD)	27.8 (4.0)	28.2 (3.3)
	History of smoking, N (%)	100 (67.6)	15 (65.2)
**Comorbidities**	Hypertension, N (%)	63 (42.6)	11 (47.8)
	Hyperlipidaemia, N (%)	42 (28.4)	8 (34.8)
	Cardiovascular disease, N (%)	60 (40.5)	10 (43.5)
	Diabetes mellitus, N (%)	18 (12.2)	2 (8.7)
**Disease severity (n = 147)**	Days of illness, mean (SD)	23.0 (17.1)	26.3 (17.6)
	Hospital care, N (%)	75 (50.7)	14 (60.9)
	Days, mean (SD)	11.0 (12.2)	14.7 (15.8)
	Intensive care, N (%)	21 (14.2)	8 (34.8)
	Days, mean (SD)	18.6 (11.9)	20.5 (13.2)
	Days recovered during follow-up visit, mean (SD) + range	628.8 (30.3) 543-690	625.3 (34.7) 562-688
**Vaccination status**	No vaccinations, N (%)	4 (2.7)	0 (0)
	1 vaccination, N (%)	2 (1.4)	0 (0)
	2 vaccinations, N (%)	20 (13.5)	1 (4.3)
	3 or more vaccinations, N (%)	122 (82.4)	22 (95.7)
**Measured biomarkers**	ET-1, vWF	+	*
	IL-18, IL-6, IL-1Ra	+	*
	Coagulation complexes	+	*
	Olink	–	+

BMI, Body Mass Index; ET-1, endothelin-1; IL, interleukin; SD, standard deviation; T0, acute phase; T1, 3 months after acute COVID-19 infection; T2, 18-24 months after acute COVID-19 infection (7); +, measurement performed; -, measurement not performed; *, measurement only performed for T1 and T2.

### High prevalence of PCC symptoms at 24 months after acute COVID-19 infection

**Figure 1:**
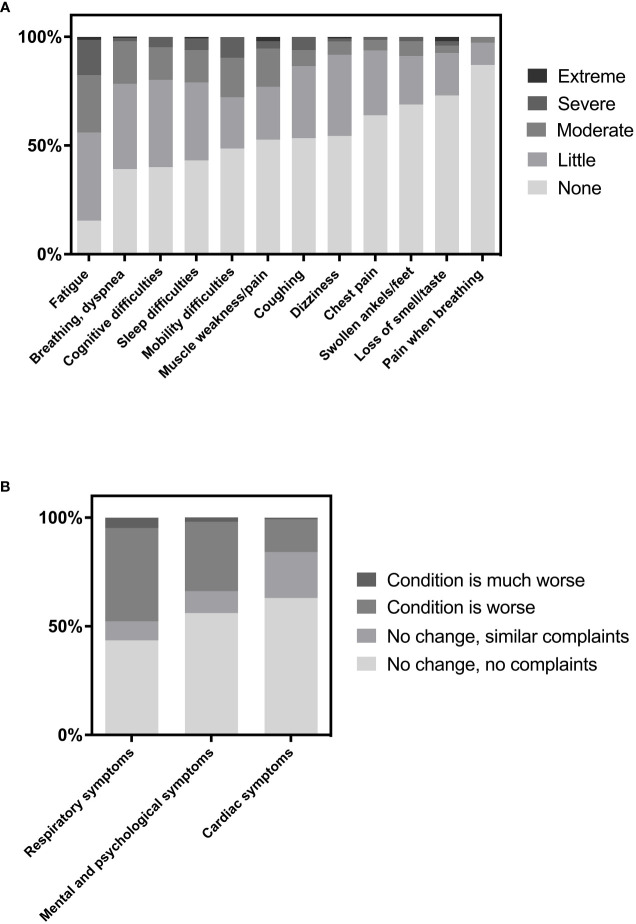
**(A)** Prevalence of post-COVID symptoms and **(B)** Perception of several conditions at 24 months after acute COVID-19 infection versus before COVID-19 infection.

At 24 months after infection, many participants still experienced PCC symptoms (**Figure 1A**). With 84.5% of participants experiencing at least some fatigue, fatigue is the most prevalent PCC symptom in this cohort. In addition, over 50% of the participants experienced problems with breathing, cognition, sleep or mobility. Loss of smell or taste was present in 27% of the participants. In total, 30.3% of participants still experienced at least one severe or extreme (level 4 or 5 on 5-point scale) PCC symptom. No significant differences in prevalence of PCC symptoms were found between patients who recovered at home, were hospitalised, and/or were transferred to the intensive care unit. Furthermore, a substantial number of participants reported worse condition of their airways, heart or mental and psychological health at 24 months after acute COVID-19 infection compared to pre-infection (**Figure 1B**).

### Plasma concentrations of biomarkers

Plasma concentrations of inflammatory cytokines IL-18 and IL-1Ra significantly decreased between 3 months and 18-24 months after COVID-19 infection, p < 0.001 and p < 0.001 respectively (**Table 2**). The percentage of patients with high levels of these inflammatory cytokines was approximately halved at 18-24 months compared to 3 months after acute COVID-19. On the contrary, a small and significant increase in plasma concentration of IL-6 was observed at long-term after COVID-19 recovery (2.9 (1.3 – 5.2) pg/mL) compared to 3 months after COVID-19 recovery (1.7 (1.0 - 3.1) pg/mL). Additionally, a larger number of participants had high levels of IL-6 (20.5 vs 3.4%).

**Table 2:** Levels of inflammatory cytokines, markers of endothelial dysfunction, and coagulation factor : inhibitor complexes at 18-24 months after acute COVID-19 infection (n=145).

	Normal range (mean ± 1 SD)	T1 (± 3 months) Mean (IQR)	High (%)	T2 (18-24 months) Median (IQR)	High, %	T1 vs T2 WSRT
Inflammatory cytokines						
IL-18	37-215 pg/mL	285.0 (212.5 – 357.1)	73.8	183.0 (135.4 – 242.3)	35.2	p < 0.001
IL-6	4.63-5.74 pg/mL	1.7 (1.0 – 3.1)	3.4	2.9 (1.3 – 5.2)	20.5	P = 0.005
IL-1Ra	100-400 pg/mL	396.0 (302.8 – 573.3)	49.2	264.4 (170.7 – 383.2)	23.4	P < 0.001
Markers of endothelial dysfunction						
ET-1	0.87-1.61 pg/mL	1.9 (1.5 – 3.1)	63.5	1.4 (1.1 – 1.8)	30.6	P < 0.001
VWF : Ag	≤ 160%	249.4 (173.9 – 350.0)	81.1	231.4 (175.9 – 317.1)	79.2	P = 0.036
Coagulation factor : inhibitor complexes						
TAT	≤ 4.0 µg/L	4.1 (3.3 – 5.1)	50.7	15.9 (9.3 – 23.9)	95.8	P < 0.001
FXIa : AT	7.0-12.5 pg/mL	11.8 (11.8 – 11.8)	17.6	11.8 (11.8 – 11.8)	4.9	P < 0.001
FXIa:a1AT	78.6-120.1 pg/mL	70.0 (70.0 – 70.0)	20.9	70.0 (70.0 – 70.0)	0.7	P < 0.001
FXIa:C1inh	176.7-396.7 pg/mL	78.8 (78.8 – 146.4)	19.6	149.1 (78.8 – 350.7)	13.5	P = 0.002
FIXa : AT	187.3-265.9 pg/mL	240.3 (223.3 – 267.1)	26.4	206.1 (188.0 – 275.5)	25.0	P = 0.003
FVIIa : AT	237.7-374.6 pg/mL	293.3 (250.0 – 453.2)	35.1	332.7 (285.0 – 420.3)	35.4	P = 0.007

IL, interleukin; IQR, interquartile range; VWF, von Willebrand factor; WSRT, Wilcoxon Signed Ranks Test.

Mean ET-1 concentrations had declined at 18-24 months after acute COVID-19 infection, and were within the normal range (**Table 2**). On the other hand, mean VWF : Ag concentrations were still elevated in 79.2% of participants suggesting endothelial dysfunction. FVIIa : AT, a marker of the extrinsic pathway, FXIa:C1inh, a marker of the intrinsic pathway, and TAT, a marker of a prothrombotic state, significantly increased between 3 and 18-24 months after infection. Levels of biomarkers did not differ between patients who recovered at home, were hospitalised, or were transferred to the intensive care unit.

### Correlations between PCC symptoms, inflammatory cytokines, markers of endothelial function and coagulation activity

**Figure 2:**
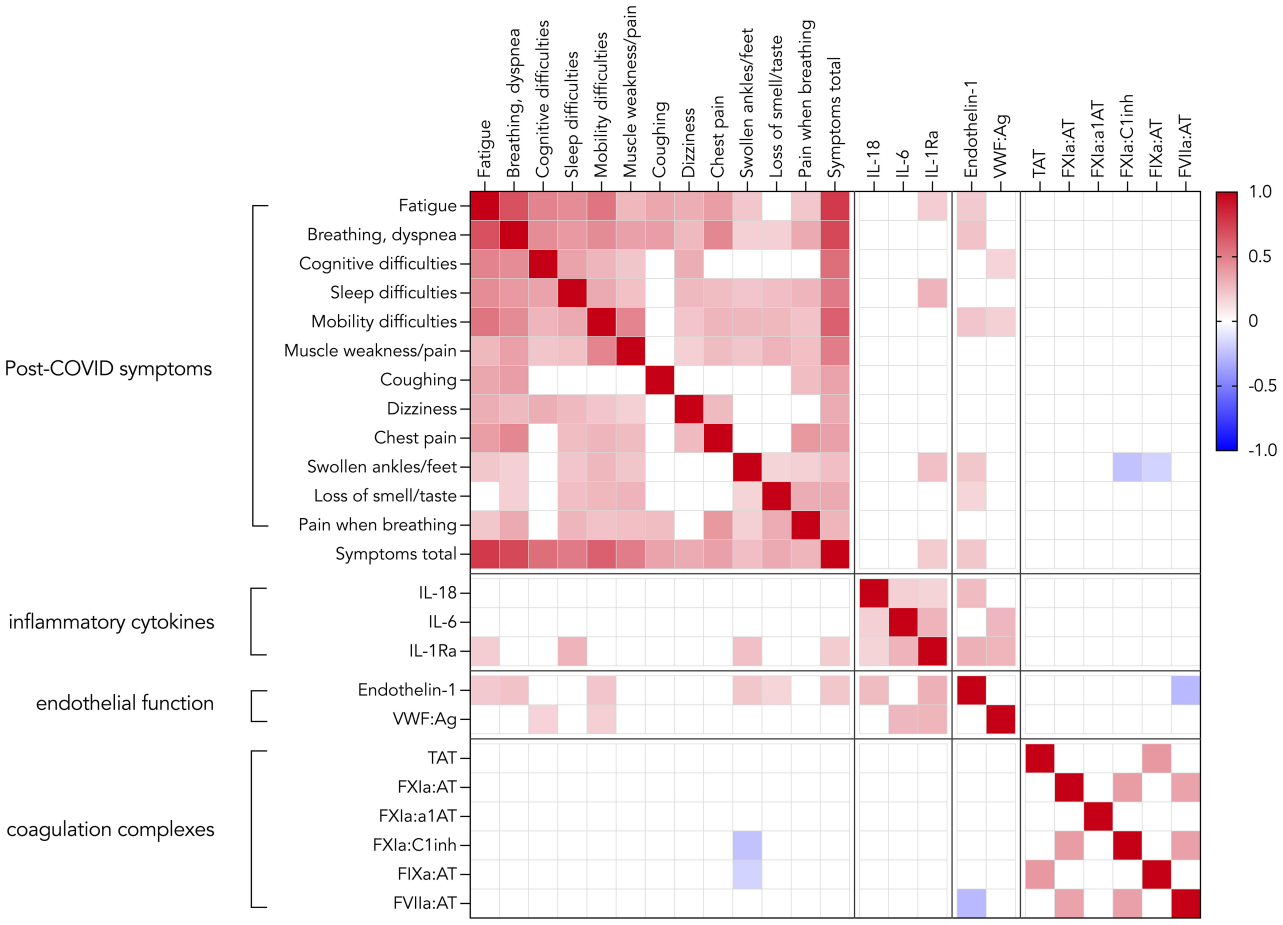
Correlation heatmap displaying Spearman’s *r* of statistically significant correlations (p < 0.05) between post-COVID symptoms, inflammatory cytokines, markers of endothelial dysfunction and coagulation factor:inhibitor complexes (n=145).

**Figure 2** shows many correlations between the various PCC symptoms reported at 24 months after acute COVID-19 infection. Breathing/dyspnea correlates with all other PCC symptoms while coughing only correlates with four other symptoms.

Furthermore, multiple weak correlations (**Supplemental Figure 2**) were found between several PCC symptoms and markers of endothelial dysfunction (ET-1 and VWF : Ag) and the inflammatory cytokine IL-1Ra. The correlations between total symptoms score and IL-1Ra and ET-1 remained significant after multivariable regression analysis (p = 0.029 and p = 0.003, respectively). Out of baseline characteristics, disease severity and comorbidities, only diabetes was a significant predictor of the total symptoms score. No correlations were found between PCC symptoms and coagulation complexes except for negative correlations between 2 markers of the intrinsic pathway (FXIA:C1inh, FIXa : AT) and the PCC symptom ‘swollen ankles’.

### Olink inflammation panel analyses during the acute phase, 3 and 24 months after SARS-CoV-2 infection

**Figure 3:**
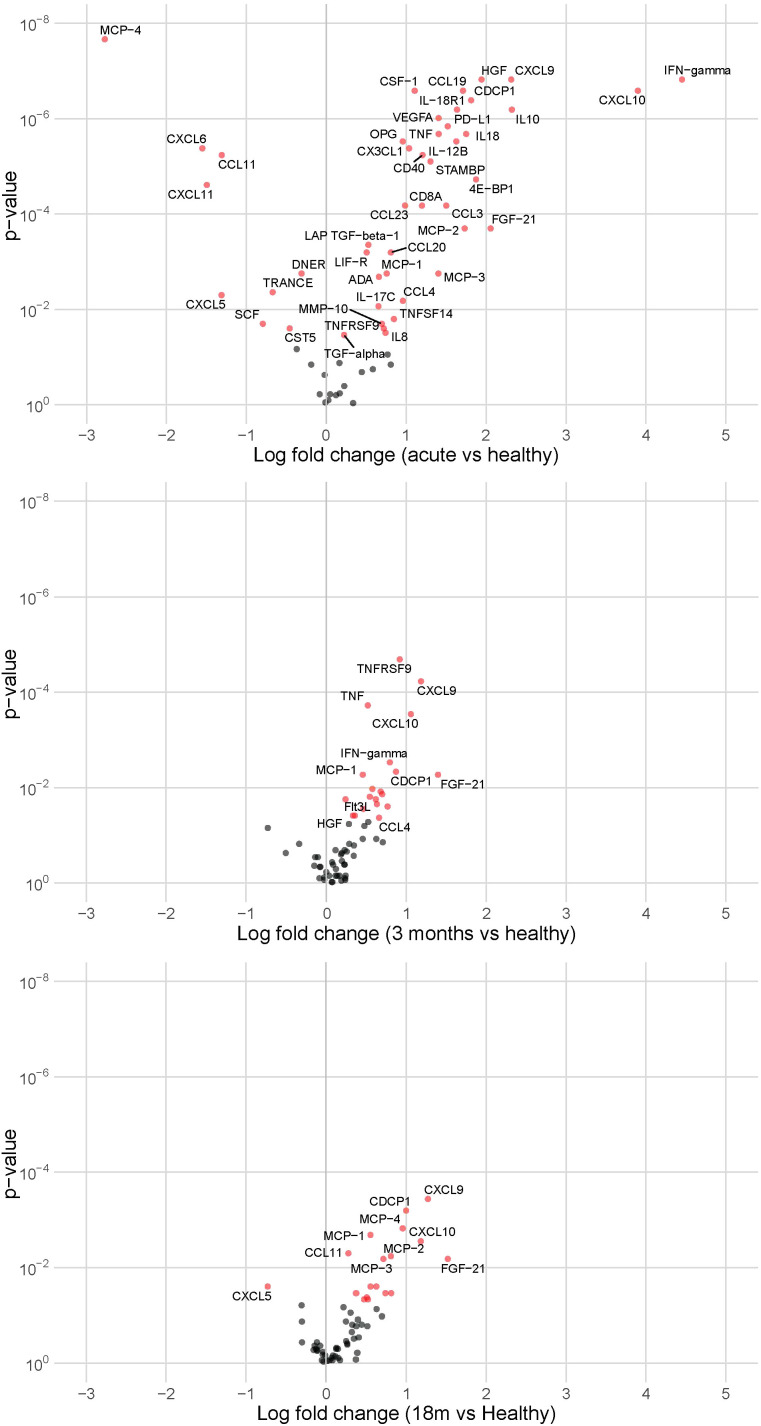
Volcano plots showing altered inflammatory proteins in our cohort (n = 23) during the acute phase, at 3 months after acute COVID-19 infection, and at 18-24 months after acute COVID-19 infection compared to healthy controls (n = 10). Red indicates significantly elevated or lowered inflammatory proteins.

Proteomics analysis of circulating inflammatory proteins in 23 PCCPCC subjects revealed that during the acute phase, many inflammatory proteins were altered compared to the healthy controls. Moreover, concentrations of CDCP1, CXCL9, CCL11, MCP-3, MCP-1, CXCL10, MCP-2, FGF-21, IL18, TNF, TNFRSF9, CCL19, CXCL11, Flt3L, IL-18R1, TRAIL, and CD40 were elevated during the acute infection and remained elevated at 3 and 18-24 months after acute COVID-19 infection (**Figure 3**), suggesting a state of chronic-inflammation. During the acute phase, multiple proteins were diminished compared to healthy controls. At 3 months after COVID-19 infection none of these proteins remained lowered, but at 18-24 months after infection CXCL5 was reduced again.

## Discussion

This study revealed a high prevalence of PCC symptoms 24 months after an acute COVID-19 infection. 84.5% of the patients experienced at least some fatigue, and over 50% suffered problems regarding breathing, cognition, sleep, or mobility. Additionally, 30.3% of the participants experienced at least one severe or extreme PCC symptom 24 months after acute infection.

According to an epidemiological update by the WHO, five SARS-CoV-2 variants of concern (VOCs) have been identified since the onset of the pandemic. The first variants of concern were described in December 2020 (14). SARS-CoV-2 emerged in the winter of 2019 from Wuhan, China (15), and the first variant with minimal genetic evolution called D614G was detected in February 2020 and became globally dominant (16, 17). Therefore, during the early phase when the patients in this cohort contracted the infection, all patients were likely infected with the original Wuhan variant or SARS-CoV-2 with the D614G mutation. Ballering et al. also described difficulties when breathing and general tiredness as core PCC symptoms due to the SARS-CoV-2 alpha variant or previous variants in people living in the Netherlands (18). They did not assess cognition and mobility, which are highly prevalent in the current study, but reported core symptoms including ageusia or anosmia, chest pain, pain when breathing and painful muscles, which we reported as PCC symptoms in the current study as well as lump in throat, heavy arms or legs, tingling extremities, and feeling hot and cold alternatively (18). Furthermore, a recent Spanish study revealed fatigue, pain, and memory loss as the most prevalent PCC symptoms 2 years after SARS-CoV-2 infection due to the Wuhan variant (19). In the Spanish cohort of hospitalised patients, 44.7% of patients experienced fatigue at 2 years after infection, but they did not specify the severity of the PCC symptoms. In the current study, 44% of patients experienced fatigue of at least moderate severity which is very similar to the prevalence of fatigue in the Spanish cohort. Additionally, in both our cohort and the Spanish cohort PCC symptoms were similar between hospitalised and non-hospitalised patients. Therefore, the prevalence of PCC symptoms in this study is in line with previous studies.

This study revealed correlations between PCC symptoms and ET-1 plasma concentrations as a marker of microvascular dysfunction, which is a potent vasoconstrictor and pro-inflammatory peptide. Under healthy conditions, production of ET-1 is small and bioavailability of nitric oxide (NO) is preserved, inducing vasorelaxation. Altered ET-1 can play a pathogenic role in vascular dysfunction and the development of cardiovascular disease by NO modulation. As a result, ET receptor antagonists could provide therapeutic benefits because they may induce increased NO bioavailability and decreased concentrations of reactive oxygen species which could in turn improve endothelial function (20). In the current study, ET-1 correlated with fatigue, breathing problems, compromised mobility, swollen ankles/feet, loss of smell/taste and the total number of PCC symptoms. Therefore, therapies counteracting ET-1 may relieve some of the PCC symptoms. Previous studies have shown that 8 weeks of aerobic or resistance exercise training in healthy young humans and 3 months of aerobic exercise training in older women induce a decrease in plasma ET-1 levels (21–23). Furthermore, improvement in NO-related vasodilation has been observed in short to medium-term exercise studies (24). It may be hypothesized that this is a novel therapeutic strategy to combat PCC symptoms.

Furthermore, this study revealed positive correlations between a large proportion of PCC symptoms at 24 months after acute COVID-19 infection and inflammation-associated cytokine IL-1Ra. Serum IL-1Ra concentrations have been positively associated with risk of cardiovascular disease (CVD) (25). In the early recovery phase after acute SARS-CoV-2 infection, 37 days after symptom onset, Zoodsma et al. detected a pro-inflammatory proteome in PCC samples (26). Our proteomics data showed that high concentrations of some inflammatory proteins were present long-term, while other inflammatory markers returned within their normal ranges.

Given the correlations between IL-1Ra and PCC symptoms and the presence of elevated protein levels in plasma samples PCC18-24 months after infection, anti-inflammatory therapies may also be beneficial for people with (severe) PCC symptoms. Filgueira et al. suggested that supervised controlled exercise could balance the inflammatory peripheral components, mitigating the inflammatory process associated with PCC and its consequences (27). In patients with severe SARS-CoV-2 infection in the acute phase, immunotherapy is the cornerstone of drug therapy (28) with an important role for anti-IL-6 treatment. Our data revealed positive correlations between PCC symptoms and IL-1Ra, but not with IL-6. Therefore, immunotherapy in patients with (severe) PCC symptoms may require targeting of different inflammatory pathways.

We did not show any association between markers of coagulation activity and symptoms, except for one distinct feature, swollen ankles, negatively linked to activation of factors XI and IX; the latter may suggest an indirect involvement of the contact system, with biased signalling of factor XIIa in the direction of inflammation through the kinin/kallikrein pathway in favour of reduced activation of factor XI and downstream intrinsic coagulation (29). The overall lack of association between coagulation and symptoms is important, given the suggestions of microvascular clotting in relation to PCC symptoms done by some investigators (30). Current data do not support such an association although the role of VWF in this regard and its impact on platelet adhesion leaves open a role for platelet hyperreactivity and platelet-leukocyte interactions as contributors to thrombo-inflammation.

To the best of our knowledge, this is the first report presenting PCC symptoms 24 months after PCR-confirmed COVID-19 infection in relation to inflammatory cytokines, endothelial dysfunction and coagulation activation. Two out of the five hypothesized mechanisms of long COVID pathogenesis as described by Davis et al. (31) were investigated. Specifically, immune dysregulation was assessed by measurement of three monocyte-derived cytokines (IL-6, IL-18 and IL-1Ra) and 96 inflammatory proteins in plasma. Blood clotting and endothelial abnormalities were evaluated by determination of concentrations of coagulation complexes, ET-1 and VWF. As only plasma markers were measured, more research focusing on immune cell phenotypes such as monocytes is warranted. Previous research already implicated that monocyte alterations continue into convalescence and correspond to specific symptoms of long COVID (32). Furthermore, long COVID patients suffering from fatigue show increased expression of inflammatory genes in monocytes (33). Additional vascular function tests such as Endo-PAT (34) could be performed for a broader insight into the effect of COVID-19 infection on endothelial function.

This study has also some limitations. First, the normal ranges for the majority of the biomarkers are arbitrary and not strongly supported by robust data in literature. Second, robust data on the prevalence of core symptoms included in the questionnaire of individuals in the era before COVID-19 is lacking. Therefore, it remains challenging to attribute symptoms to PCC compared to comorbidities. Furthermore, the inclusion method and the drop-out of participants between the first measurement and the completion of the questionnaire may have increased the chance of selection bias. Individuals who are interested in the concept of PCC or who suffer from PCC symptoms may have been more likely to participate in this study. Lastly, a major limitation is that Olink analysis was performed in a relatively small subgroup of 23 participants of whom plasma was also collected during the acute and early-recovery phase of the COVID-19 infection.

In summary, this study shows that at 24 months after acute COVID-19 infection, patients still experience a high prevalence of PCC symptoms which correlate with inflammatory cytokine IL-1Ra and markers of endothelial dysfunction, especially ET-1. Our data may provide a rationale for the selection of treatment strategies for further clinical studies.

## Data availability statement

The raw data supporting the conclusions of this article will be made available by the authors, without undue reservation.

## Ethics statement

The studies involving human participants were reviewed and approved by Regional ethics committee Arnhem-Nijmegen (NL74101.091.20) and medical research ethics committee (MREC) of Maastricht University Medical Center+ and Maastricht University (METC2021-2990). The studies were conducted in accordance with the local legislation and institutional requirements. The participants provided their written informed consent to participate in this study.

## Author contributions

LW, SK, CG-D, and MCW contributed to the concept of the study. LJ, MW, LW, LG, MaN, SK, CG-D, AP and MCW contributed to the trial design. LW, MW, LJ, MaN, and AP contributed to the data collection. LJ, LW, LG, HC, and MCW contributed to the data analysis. All authors contributed to the interpretation of the data. LJ and MCW wrote the first draft of the manuscript. All authors read, revised and approved the final manuscript.
