# Supplementary material for: Biomarkers of sustained systemic inflammation and microvascular dysfunction associated with post-COVID-19 condition symptoms at 24 months after SARS-CoV-2-infection
Source: Front Immunol. 2023 Oct 5;14:1182182. doi: 10.3389/fimmu.2023.1182182 (PMC10586198; doi:10.3389/fimmu.2023.1182182)
Supplement: Supplementary file 1 [file DataSheet_1.pdf]

Supplementary Material

Biomarkers of sustained systemic inflammation and microvascular dysfunction associated with post-COVID symptoms at 24 months after SARS-CoV-2-infection

Lotte MC Jacobs MSc[1], Marieke SJN Wintjens MD[2,3], Magdolna Nagy PhD[4,5], Loes Willems MD[1], Hugo ten Cate MD PhD FAHA[4,5,6], Henri MH Spronk MD PhD[4,5], Sander M.J. van Kuijk PhD[2], Chahinda Ghossein-Doha MD PhD[5,7], Mihai G. Netea[8,9], Laszlo A Groh PhD[10†], André S. van Petersen MD PhD FEBVS [11†], Michiel C. Warlé MD PhD FEBVS[1†*]

† These authors contributed equally to this work and share senior authorship

*** Correspondence:**

M.C. Warlé, MD, PhD, FEBVS

Michiel.Warle@radboudumc.nl

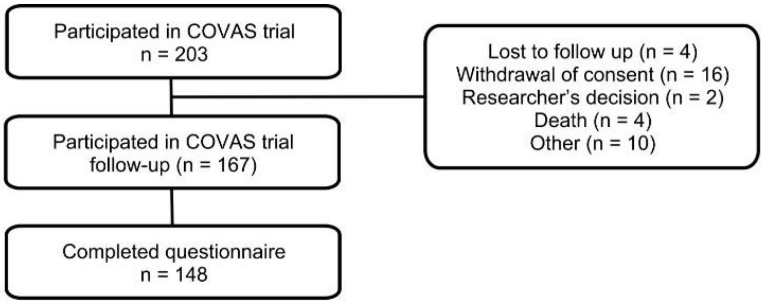

Supplementary Figure 1. Flow chart of the study population.

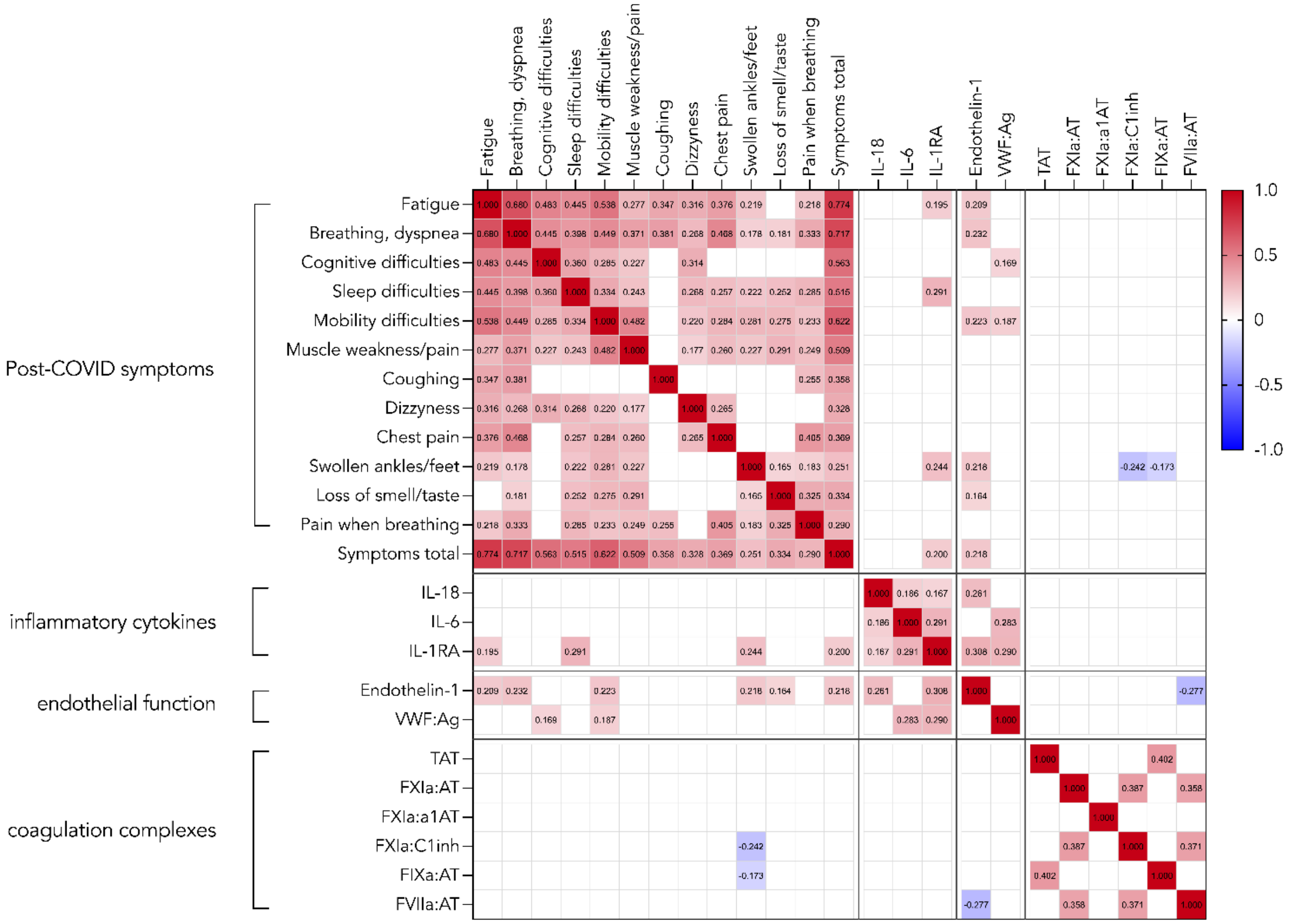

Supplementary Figure 2. Correlation heatmap displaying Spearman's r of statistically significant correlations ($p < 0.05$) between post-COVID symptoms, inflammatory cytokines, markers of endothelial dysfunction and coagulation factor:inhibitor complexes (n=145).
